# Pharmacological but Not Physiological Levels of GDF15 and FGF21 Regulate Body Weight and Glycemic Control in Obese Mice

**DOI:** 10.1096/fj.202501350R

**Published:** 2025-08-11

**Authors:** Sarah Engelbeen, Julie Mie Jacobsen, Luisa Deisen, Elisa Parsche, Alberte Buch‐Ramussen, Christoffer Clemmensen, Rune Ehrenreich Kuhre, Kornelia Johann, Maximilian Kleinert

**Affiliations:** ^1^ Department of Molecular Physiology of Exercise and Nutrition German Institute of Human Nutrition (DifE) Potsdam‐Rehbruecke Nuthetal Germany; ^2^ Global Drug Discovery Måløv Denmark; ^3^ Novo Nordisk Foundation Center for Basic Metabolic Research, Faculty of Health and Medical Sciences University of Copenhagen Copenhagen Denmark; ^4^ Department of Biomedical Sciences University of Copenhagen Copenhagen Denmark; ^5^ German Center for Diabetes Research (DZD) Munich Germany; ^6^ Institute of Nutritional Sciences University of Potsdam Nuthetal Germany

**Keywords:** diet‐induced obesity, energy homeostasis regulation, FGF21, GDF15, gene knockout mice, glucose tolerance, pharmacological weight‐loss therapy

## Abstract

GDF15 and FGF21 are stress‐induced hormone‐like factors with putative roles in the regulation of energy homeostasis. Since their plasma levels increase with obesity, it has been proposed that GDF15 and FGF21 jointly impose a cap on weight gain during diet‐induced obesity. To test this hypothesis, we generated single *Gdf15* knockout (KO) and *Fgf21* KO, and double *Gdf15/* *Fgf21* KO mice. Depletion of both GDF15 and FGF21 had minimal effects on the gain of body weight, fat, and fat‐free mass in male or female mice fed either chow diet or high‐fat, high‐sucrose diet. Similarly, glucose tolerance, fasting glucose, and plasma insulin levels were largely unaffected by the combined absence of GDF15 and FGF21. Thus, combined deletion of endogenous *Gdf15* and *Fgf21* exerted a limited influence on body weight gain or glycaemic control. By contrast, pharmacological dosing of obese male mice with long‐acting recombinant GDF15 or FGF21 produced meaningful weight loss on their own (8%–10%), and GDF15 + FGF21 co‐administration yielded an impressive, additive weight reduction of 25%. Combinatorial treatment also improved glucose tolerance, lowered fasting insulin levels, and reduced hepatic fat content. In conclusion, while endogenous GDF15 and FGF21 appear largely nonessential for the regulation of weight gain and glycemia, pharmacological co‐treatment with GDF15 and FGF21 elicits robust weight‐loss benefits.

## Graphical abstract

GDF15 and FGF21: Endogenous vs. pharmacological effects on body weight in mice.

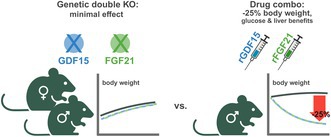

## 1 Introduction

Growth differentiation factor 15 (GDF15) and fibroblast growth factor 21 (FGF21) are stress‐responsive hormone‐like factors with putative overlapping roles in the regulation of energy balance. In support, increasing circulating levels of either GDF15 or FGF21, by injections or via genetic overexpression, robustly lowers body weight in mice and rats [1, 2, 3, 4, 5, 6, 7].

Meanwhile, the physiological function of endogenous GDF15 and FGF21 in the regulation of body weight remains uncertain. Plasma concentrations of both are typically increased in diet‐induced obesity (DIO) rodent models [8], but the increase is markedly less compared to the increase during pathophysiological states or following pharmacotherapy. To illustrate this point, in male mice fed a high‐fat diet for 16 weeks, GDF15 levels have been reported to reach approximately 300 pg/mL, which is about 3‐fold more than in corresponding chow‐fed control mice [9]. Injection with recombinant GDF15 at a dose required to reduce food intake results in peak plasma levels at around 50 000 pg/mL [9], which, clearly, is far from the physiological range and reflects pathological states such as cancer cachexia [10], with the key difference being that the levels during cancer are chronically elevated.

Nevertheless, it has been hypothesized that the higher levels of either GDF15 or FGF21 during DIO limit excessive weight gain. This would imply that the deletion of either gene should accelerate and augment weight gain during DIO. The evidence from genetic models regarding this question is mixed. While some studies observe a modest increase in weight gain during DIO upon deletion of *Gdf15* [11] or *Fgf21* [12], this is not a consistent finding [13, 14, 15, 16]. Overall, these mixed data might indicate that endogenous GDF15 or FGF21 only play a modest role in body weight regulation in response to DIO. *Gdf15* and *Fgf21* expression are regulated by similar underpinning molecular mechanisms, and circulating levels frequently increase in tandem during the development of obesity [9]. Moreover, pharmacological studies have shown that GDF15 and FGF21 have functional overlap as they both reduce energy intake and increase energy expenditure [15, 17, 18], improve glycemia [15] and decrease body weight of DIO mice or rats [2, 15]. Similarly, chronic over‐expression of either GDF15 or FGF21 alone protects against DIO [19]. Based on their similar regulation and overlapping functions, it is possible that genetic deletion of one factor is functionally compensated for by the other, thereby masking a role of each in the regulation of body weight and glycemic control.

To test the hypothesis, we deleted both *Gdf15* and *Fgf21* in male and female mice. We chose to investigate both sexes, since sex‐dependent effects for cytokines on metabolic outcomes have been reported [20]. We assessed body weight and glycemia during chow and high fat, high sucrose (HFHSD) feeding. We found that endogenous GDF15 and FGF21 are largely nonessential for the regulation of body weight and glycemia in normal weight chow‐fed mice and in DIO mice. In contrast to this, mono‐therapy with long‐acting recombinant GDF15 or FGF21 resulted in relevant weight losses, and combination therapy (GDF15+FGF21) gave rise to a robust (≈25%) additive weight loss in male DIO mice.

## 2 Material and Methods

### 2.1 Animal Breeding and Housing

Global *Gdf15*‐knockout (Gdf15^−/−^) mice were first established on a mixed C57BL/6 × 129/SvJ background [21], and global *Fgf21*‐knockout (Fgf21^−/−^) mice were generated from 129/Sv ES‐cell chimeras bred with C57BL/6 females as described previously [22]. To generate *Gdf15* and *Fgf21* double knockout (KO) mice for the metabolic characterization studies, we first bred *Gdf15* KO (*Gdf15* ^−/−^) mice with *Fgf21* KO (*Fgf21* ^−/−^) mice to obtain the first generation of mice heterogeneous for *Gdf15* and *Fgf21* (*Gdf15* ^+/‐^x*Fgf21* ^+/−^). From this generation onwards, all mice were bred from *Gdf15* ^+/‐^x*Fgf21* ^+/−^ mice. The resulting wildtype (WT, *Gdf15* ^+/+^x*Fgf21* ^+/+^), *Gdf15* KO (*Gdf15* ^−/‐^x*Fgf21* ^+/+^), *Fgf21* KO (*Gdf15* ^+/+^x*Fgf21* ^−/−^) mice, and *Gdf15*x*Fgf21* double KO (*GF* dKO, *Gdf15* ^−/‐^x*Fgf21* ^−/−^) mice were used for the metabolic characterization. Mice were housed in individually ventilated cages (IVC) at a temperature of 23°C ± 1°C and with ad libitum access to food and drinking water. At 3 weeks of age, male and female mice were weaned and housed in groups of two mice per cage. All procedures were reviewed and approved by the Landesamt for Verbraucherschutz and Gesundheit (LAVG) Brandenburg, Germany under the registration AZ2347‐14‐2021.

For the FGF21 and GDF15 mono‐ and combination therapy study, DIO male C57BL/6J mice were purchased at Charles River (France) at 20 weeks of age. Upon arrival to the animal facility (Måløv, Novo Nordisk), mice were single‐housed in a standard type IV cage with a transparent plastic divider and with bedding and a plastic tunnel. Mice had ad libitum access to 45% HFD (Cat. No. D12451, Research Diet, NJ, USA) and followed a 12:12 light: dark rhythm with lights on at 0600‐1800 h. Food was replaced daily.

### 2.2 Mouse Studies

#### 2.2.1 Metabolic Characterization Studies

In the metabolic characterization studies, male and female WT, *Gdf15* KO, *Fgf21* KO, and *GF* dKO *21* ^−/−^) mice were fed either a standard chow (V1534‐703, ssniff Spezialdiäten GmbH, Germany) or high fat–high sucrose diet (HFHSD, EF D12331, ssniff Spezialdiäten GmbH, Germany) with a fat, protein, and carbohydrate content of 59 kJ%, 15 kJ%, and 25 kJ%, respectively, including 17% sucrose by weight.

In the chow‐fed cohort, body weight and food intake were determined once a week from the age of 3 weeks. From 10 weeks of age, body composition was measured by nuclear magnetic resonance spectroscopy using the EchoMRI (EchoMRI LLC) every 2 weeks. Glucose tolerance was determined at 12 weeks of age. Mice were sacrificed at 18 weeks of age, and blood was collected via cardiac puncture, and tissues were isolated, frozen in liquid nitrogen, and stored at −80°C for molecular analysis.

For the HFHSD‐fed cohort, mice were fed, ad libitum, with the HFHSD from the age of 8 weeks. Body weight was measured every week and body composition was determined by NMR every 4 weeks. After 14 weeks on HFHSD, mice underwent a glucose tolerance test (GTT). In male mice, energy expenditure was determined by indirect calorimetry (Phenomaster, TSE Systems, Germany) after 18 weeks on HFHSD. All mice were sacrificed at the age of 28 weeks, after being fed with HFHSD for a total of 20 weeks, and blood and tissues were collected for post‐mortem analysis.

##### 2.2.1.1 Glucose Tolerance Tests

Mice were fasted for 6 h from the onset of the light phase. At the end of the fasting period, an initial blood sample was collected from the tail to measure basal blood glucose levels with a handheld glucometer (CONTOURNEXT from Bayer) and insulin levels by ELISA (Mouse Ultrasensitive Insulin ELISA, Alpco, Cat#80‐INSMSU‐E01, E10). Then, mice received an intraperitoneal injection of 1.5 g (HFHSD‐fed mice) or 2 g (chow‐fed mice) glucose per kg body weight with an injection volume of 10 mL per kg body weight, and blood glucose was measured in tail vein blood using handheld glucometers (CONTOURNEXT, Bayer) 15, 30, 60, and 120 min after injection.

##### 2.2.1.2 Indirect Calorimetry

Energy expenditure and basal metabolic rate were determined in male HFHSD‐fed mice using the TSE Phenomaster System. Mice were single‐housed in TSE cages for 3 days for acclimatization to the new environment, as well as feeding and drinking systems. Afterwards, daily energy expenditure was measured inside the TSE Phenomaster at 23°C ± 1°C for 3 days. On day 4, food was withdrawn 1 h after the onset of the light phase, and the temperature of the Phenomaster climate chamber was raised to 30°C for the measurement of basal metabolic rate at thermoneutrality. After 5 h, the temperature was decreased to 23°C ± 1°C, and food access was restored.

##### 2.2.1.3 Measurement of Plasma GDF15 and FGF21 Levels

Plasma levels of GDF15 (Mouse/Rat GDF‐15 Quantikine ELISA Kit, MGD150, R&DSystems Inc.) and FGF21 (Mouse/Rat FGF‐21 Quantikine ELISA Kit, MF2100, R&DSystems Inc.) were determined according to the manufacturer's instructions.

##### 2.2.1.4 Determination of Triacylglycerol (TG) Levels in Plasma and Liver

Plasma and liver TG were determined using the TG Determination Kit (Sigma‐Aldrich). Liver TG was measured after extraction with 10 mmol/L sodium phosphate buffer (pH 7.4) containing 1 mmol/L EDTA and 1% polyoxyethylene [10] tridecyl ether.

##### 2.2.1.5 Gene Expression Analysis

In the metabolic characterization studies, total RNA was isolated from liver with TriFast (30‐2010P, VWR) according to the manufacturer's instructions. Genomic DNA was digested with DNase I (EN0521, Fisher Scientific) for 30 min. at 37°C, in the presence of Ribolock RNase inhibitor (EO0382, Fisher Scientific), after which DNase I was deactivated by heating at 65°C for 10 min. Then, cDNA was prepared with 1 μg of RNA and LunaScript RT Super Mix (E3010L, New England Biolabs). Expression levels of genes associated with hepatic lipid oxidation and lipogenesis were assessed with quantitative PCR (qPCR). qPCRs were performed in triplicates per sample in the presence of forward and reverse primers (Table 1) and Luna Universal qPCR Master Mix (M3003E, New England Biolabs) on a VIA7 Real Time PCR System (4 453 534, Thermo Fischer Scientific). Primer efficiency was determined with an experiment‐specific standard curve and target gene expression was normalized to the expression levels of appropriate housekeeping genes (Hprt).

**TABLE 1:** List of primers for RT‐qPCR and PCR.

Gene	Forward primer 5′‐3′	Reverse primer 5′‐3′
*Fgf21*	CCTCTAGGTTTCTTTGCCAACAG	AAGCTGCAGGCCTCAGGAT
*Gdf15*	CCGAGAGGACTCGAACTCAG	GGTTGACGCGGAGTAGCAG
*Cd36*	ATGGGCTGTGATCGGAACTG	GTCTTCCCAATAAGCATGTCTCC
*Cpt1a*	CTCCGCCTGAGCCATGAAG	CACCAGTGATGATGCATTCT
*Acadl*	ATTGCTGAGTTGGCGATTTC	GCTGCACCGTCTGTATGTGT
*Acaca*	ATGGGCGGAATGGTCTCTTTC	TGGGGACCTTGTCTTCATCAT
*Dgat2*	GCGCTACTTCCGAGACTACTT	GGGCCTTATGCCAGGAAACT
*Elovl6*	GAAAAGCAGTTCAACGAGAACG	AGATGCCGACCACCAAAGATA
*Pparα*	AGAGCCCCATCTGTCCTCTC	ACTGGTAGTCTGCAAAACCAAA
*Scd1*	TTCTTGCGATACACTCTGGTGC	CGGGATTGAATGTTCTTGTCGT
*Fasn*	GGAGGTGGTGATAGCCGGTAT	TGGGTAATCCATAGAGCCCAG
*Hprt*	TCAGTCAACGGGGGACATAAA	GGGGCTGTACTGCTTAACCAG

#### 2.2.2 Pharmacological Treatment in DIO Mice

DIO mice purchased at age 20 weeks were fed ad libitum with a moderate high‐fat, high‐sucrose diet (D12451, Research Diet) with a total fat, protein, and carbohydrate content of 45 kJ%, 20 kJ%, and 35 kJ%, respectively, including 20% sucrose by weight. Mice were fed this diet for 5 months prior to the study. Eight days prior to the first injection, mice were weighed and randomly allocated to treatments while balancing groups for similar body weight. Baseline body composition was determined with the E56‐070 EchoMRI scanner 3 days prior to the start of daily injections. On day 0, mice received daily subcutaneous injections (5 mL/kg) of recombinant GDF15 (0.4 nmol/kg, NNC0247‐0000‐0880), recombinant FGF21 (5 nmol/kg, NNC0194‐0000‐0001), or rGDF15 + rFGF21 (0.4 + 5 nmol/kg), between 10.00 and 11.00 am each day for 24 days. Body weight and food intake were measured daily. Body composition was assessed again on day 22 with the E56‐070 EchoMRI scanner. A glucose tolerance test was performed on day 24. Animals were terminated by isoflurane overdose on day 26 (09.00–11.30 h) 3 h after the last injection, and during this time mice were without food. Blood was collected into EDTA‐coated Eppendorf tubes, centrifuged (5 min, 4°C, 6000 rpm) and plasma was transferred into fresh Eppendorf tubes and stored at −20°C until analysis. Liver tissue (30–70 mg) was obtained for quantification of liver triacylglycerol (TG). This piece of tissue was snap frozen on dry ice and was stored at −80°C before analysis. Liver TG and plasma TG concentrations, as well as plasma activity of aspartate aminotransferase and alanine aminotransferase (ALT), were quantified on a Cobas 6000 analyzer (F. Hoffmann‐La Roche AG, Basel, Switzerland), following instructions provided by the manufacturer.

##### 2.2.2.1 Glucose Tolerance Test

Mice were fasted for 5 h from the onset of the light phase. At the end of the fasting period, an initial blood sample was collected from the tail to measure basal blood glucose levels with a handheld glucometer (Biosen EKF diagnostics). Then, glucose was injected intraperitoneally at 1.5 g glucose/kg body weight with an injection volume of 7.5 mL/kg and blood glucose was measured in tail vein blood using handheld glucometers (Biosen EKF diagnostics) 15, 30, 60, and 120 min after injection.

#### 2.2.3 Statistics

Data were analyzed with Graphpad Prism (Graphpad Software, San Diego, CA, version 10.2.3). Values are presented as means ± standard error of the mean. A *p*‐value of < 0.05 is considered significant. The type of statistical testing performed is described in the figure legends. Differences in energy expenditure were calculated using ANCOVA with body weight as covariate using SPSS (version 28).

#### 2.2.4 Data and Resource Availability

No large datasets were generated. The data generated and analyzed during the current study are available from the corresponding author upon reasonable request.

## 3 Results

### 3.1 Endogenous Levels of FGF21 and GDF15 Are Dispensable for the Regulation of Body Weight and Glycemia in Chow‐Fed Mice

**FIGURE 1:**
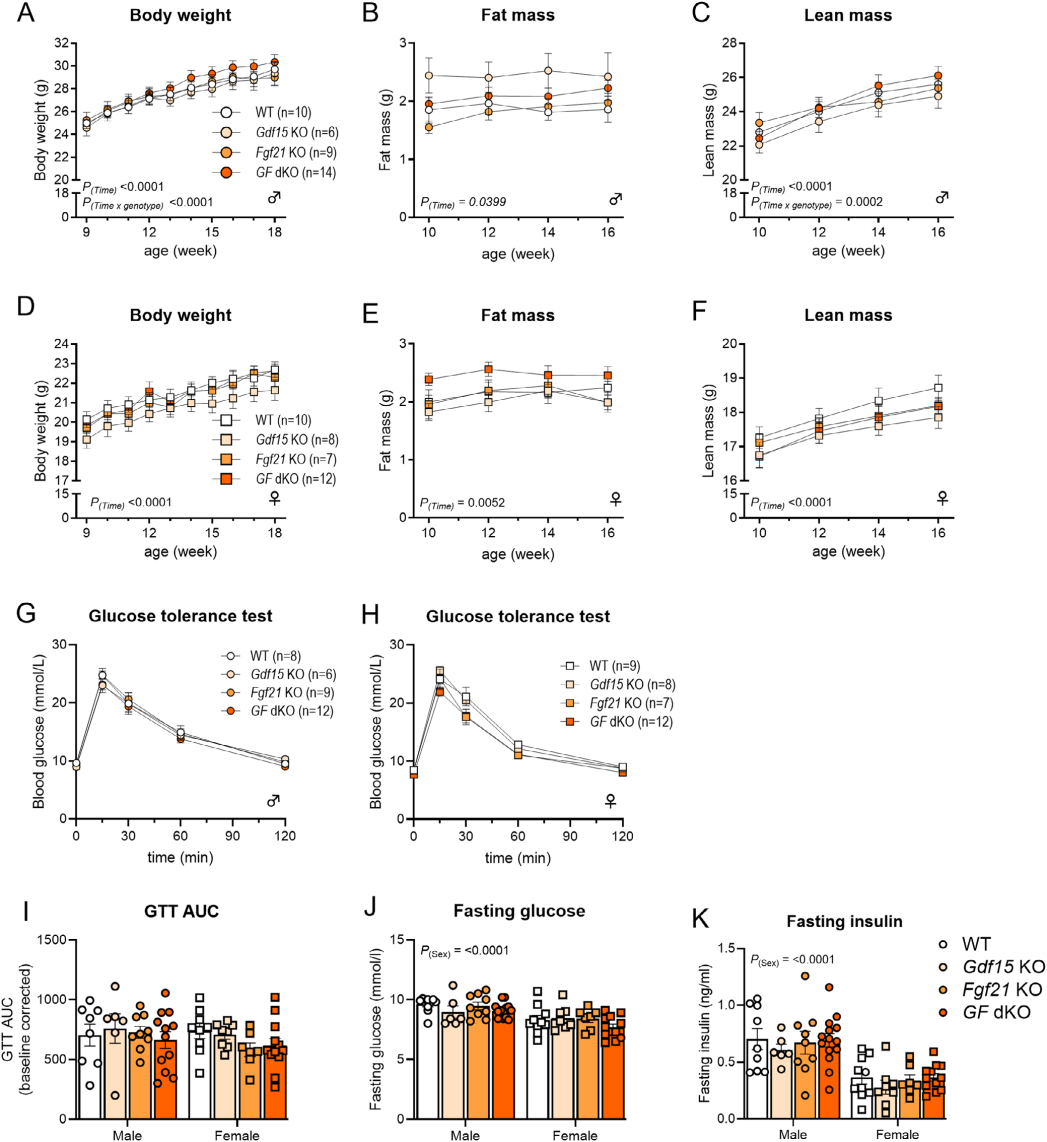
Endogenous GDF15 and FGF21 are dispensable for regulation of body weight in male and female chow‐fed mice. (A–F) Body weight, fat mass and lean mass in chow‐fed male (A–C) and female (D–F) wildtype (WT), *Gdf15* knockout (KO), *Fgf21* KO, and *Gdf15 × Fgf21* (*GF*) double KO (dKO). (G, H) Glucose tolerance curves for male (G) and female (H) mice determined at age 12 weeks and corresponding (I) baseline‐corrected areas under the curve. (J) Fasting glucose and (K) fasting insulin levels prior to glucose tolerance test. Statistic: (A–H) two‐way (genotype × time) repeated measures ANOVA with Tukey post hoc testing when an interaction was detected; (I–K) two‐way (genotype × sex) ANOVA.

We characterized chow‐fed male and female wild‐type (WT), *Gdf15* KO, *Fgf21* KO, and *Gdf15/Fgf21* (*GF*) dKO mice from 9 to 18 weeks of age. In male mice, body weight, fat mass, and fat‐free mass increased over time (Figure 1A–C). We found a statistical interaction that indicated differential development of body weight and fat‐free mass among genotypes over time; however, post hoc analyses did not reveal differences among genotypes at specific time points (Figure 1A,C). In female mice, body weight, fat mass, and fat‐free mass increased to a similar extent across all genotypes (Figure 1D–F). Glucose tolerance was similar across all groups in both male and female mice (Figure 1G–I). Female mice had lower fasting glucose and insulin levels than male mice (Figure 1J,K).

Plasma GDF15 and hepatic *Gdf15* mRNA levels were undetectable in *Gdf15* KO and *GF* dKO mice, but expression levels were similar in WT and *Fgf21* KO mice. In female mice, circulating GDF15 levels were generally lower, whilst hepatic *Gdf15* mRNA levels were higher (Figure S1A,D). Plasma FGF21 and hepatic *Fgf21* mRNA levels were undetectable in *Fgf21* KO and *GF* dKO mice. In male *Gdf15* KO mice, plasma FGF21 and hepatic *Fgf21* mRNA levels were similar to those in WT mice. In female *Gdf15* KO mice, plasma FGF21 levels and hepatic *Fgf21* mRNA abundance were lower compared to WT mice (Figure S1B,E).

Plasma and liver triacylglycerol (TG) did not differ among groups. Female mice had lower circulating TG levels but higher hepatic TG levels than male mice (Figure S1C,F). Expression levels of genes associated with hepatic lipid oxidation and lipogenesis were consistently higher in females but did not depend on genotype (Figure S1G–O).

### 3.2 Endogenous Levels of FGF21 and GDF15 Are Largely Dispensable for the Regulation of Body Weight and Glycemia in High‐Fat and High‐Sucrose Diet‐Fed Mice

**FIGURE 2:**
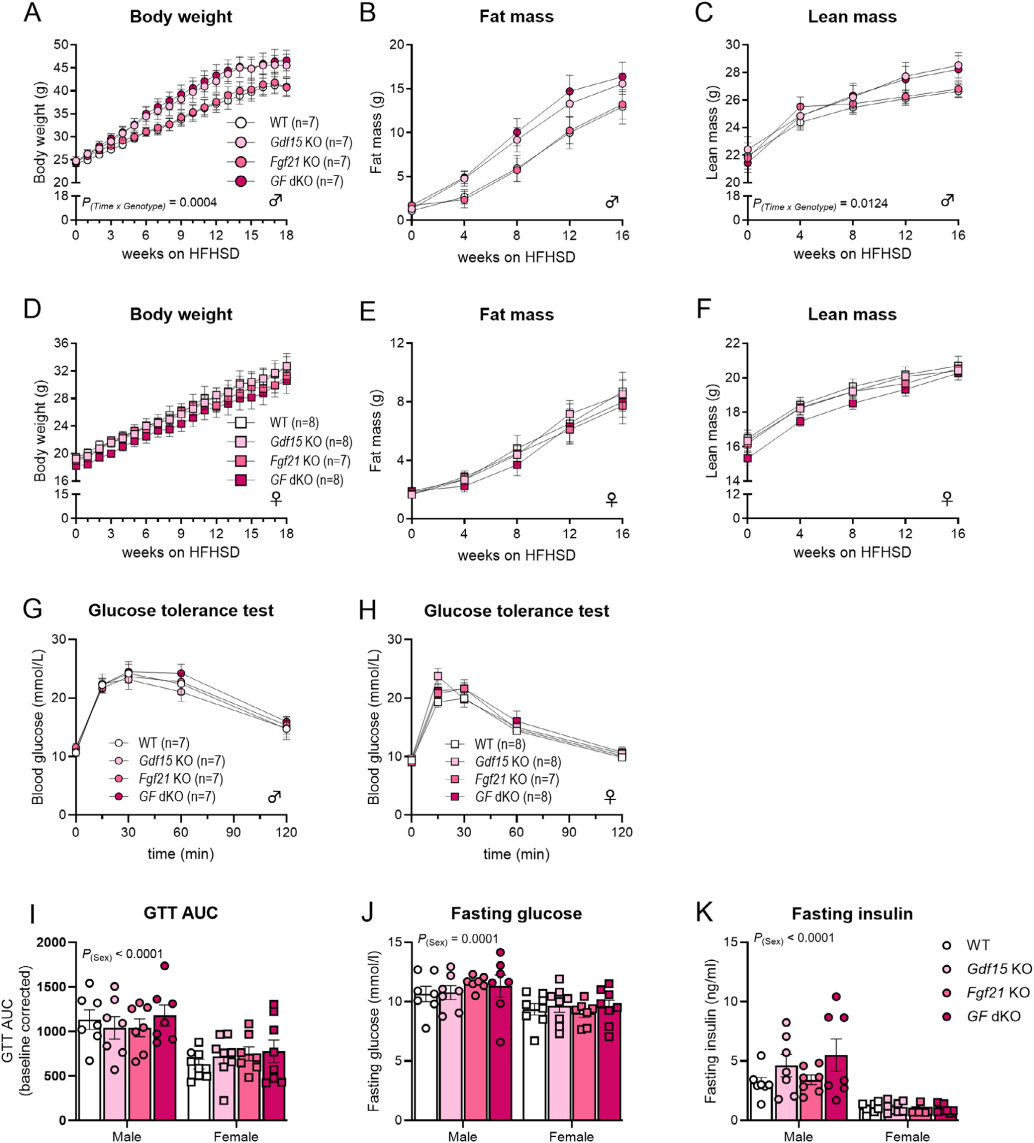
Body weight and glucose tolerance is not influenced by loss of endogenous GDF15 and FGF21 in HFHSD‐fed mice. Body weight, fat mass, and lean mass were recorded over time in male (A–C) and female (D–F) mice, fed a high‐fat high‐sucrose diet (HFHSD) starting at 8 weeks of age (defined as 0 weeks on HFHSD); Groups include Wildtype (WT), *Gdf15* knockout (Gdf15 KO), *Fgf21* knockout (Fgf21 KO), and double knockout (dKO) of *Gdf15* and *Fgf21* (*GF* dKO). (G, H) Glucose tolerance curves for male (G) and female (H) mice determined in week 14 on the HFHSD and corresponding (I) baseline‐corrected areas under the curve. (J) Fasting glucose and (K) fasting insulin levels prior to glucose tolerance test. Statistic: (A–H) two‐way (genotype × time) repeated measures ANOVA with Tukey post hoc testing when an interaction was detected; (I–K) two‐way (genotype × sex) ANOVA.

To investigate whether GDF15 and FGF21 limit weight gain during DIO, male and female WT, *Gdf15* KO, *Fgf21* KO, and *GF* dKO mice were fed a high‐fat, high‐sugar diet (HFHSD) for 20 weeks, starting at 8 weeks of age. Male mice increased body weight, fat mass, and fat‐free mass during this period (Figure 2A–F). Similar to chow‐fed male mice, a significant interaction indicated differential development of body weight and fat‐free mass among genotypes over time; post hoc testing failed to detect differences among genotypes (Figure 2A–C). In female mice, body weight, fat mass, and fat‐free mass increased to a similar extent across all groups (Figure 2D–F).

Female mice were more glucose tolerant than male mice, but within sex, glucose tolerance was similar among genotypes (Figure 2G–I). HFHSD‐fed female mice had overall lower fasting glucose and insulin levels than males. In male mice, fasting insulin levels were elevated in *GF* dKO mice compared to WT mice (Figure 2K).

**FIGURE 3:**
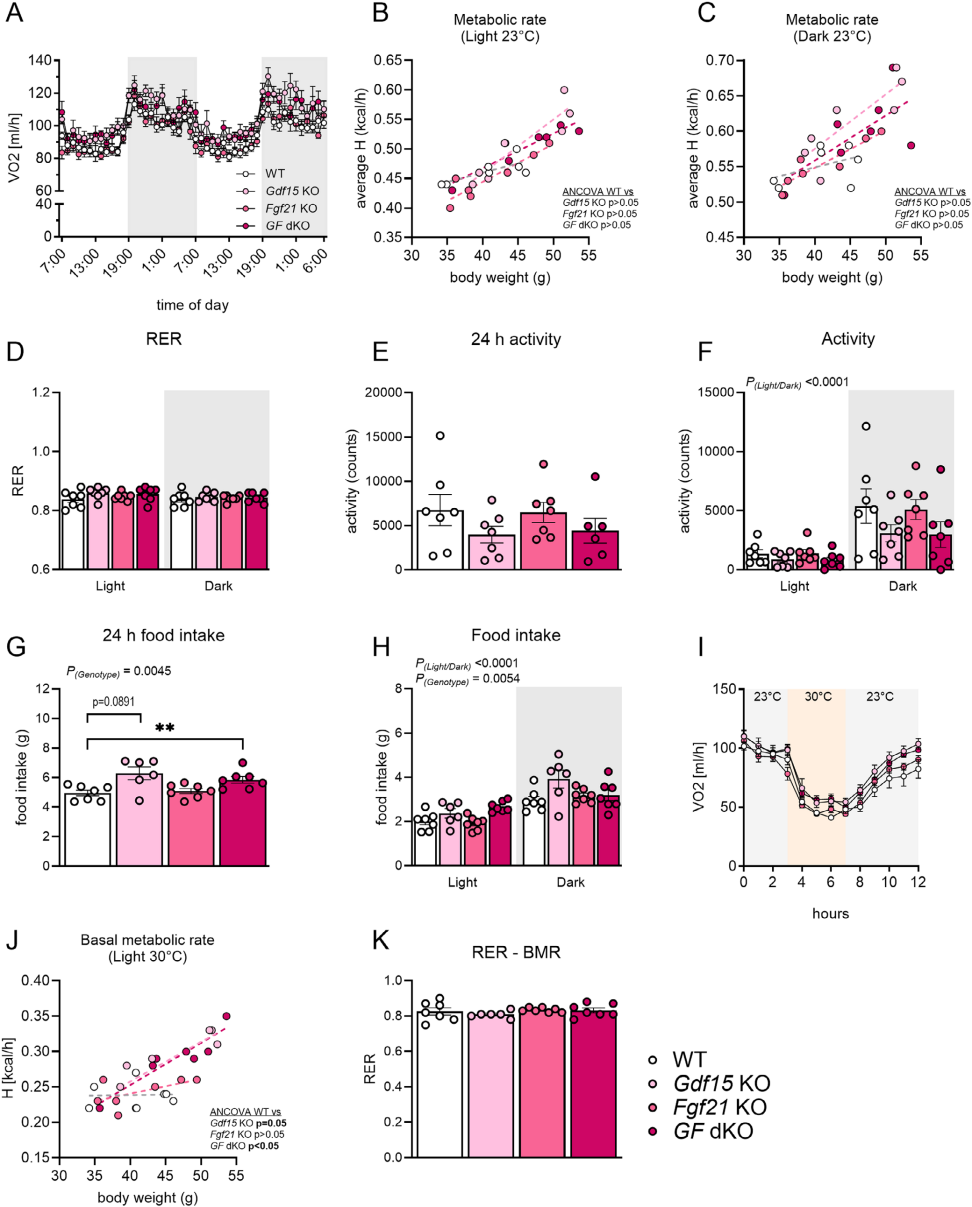
Endogenous GDF15 and FGF21 are dispensable for regulation of total energy expenditure. Energy expenditure, food intake, and substrate utilization in male HFHSD‐fed mice determined in week 18 on the HFHSD (see legends of Figure 2). Groups include Wildtype (WT), *Gdf15* knockout (Gdf15 KO), *Fgf21* knockout (Fgf21 KO), and double knockout (dKO) of *Gdf15* and *Fgf21* (*GF* dKO). Total oxygen consumption (A); and metabolic rate as a function of body weight during light (B) and dark (C) phase. Respiratory exchange ratio (RER) (D), activity (E, F), and food intake (G, H). Oxygen consumption (I) during determination of basal metabolic rate (J) at 30°C and corresponding RER (K). Statistic: (B, C, and J) ANCOVA with body weight as co‐variate; (F, H) two‐way (genotype × phase) ANOVA; (G) one‐way ANOVA with Tukey post hoc testing. **p < 0.01 for difference between indicated groups.

Given the statistical interaction between genotype and time, and a difference of 4–5 g in body weight between male *Gdf15* KO (45.64 ± 2.07 g) or *GF* dKO (46.43 ± 2.59 g) mice to WT (41.229 ± 1.88 g) mice after 17 weeks on the HFHSD, we investigated potential genotypic differences in food intake and energy expenditure at week 18. Total energy expenditure (Figure 3A–C), substrate utilization (Figure 3D) and spontaneous activity were similar (Figure 3E,F) across the groups. Food intake was higher in *GF* dKO mice and trended (*p* = 0.0891) to be higher in *Gdf15* KO mice (Figure 3G,H). Basal metabolic rate was higher in *Gdf15* KO and *GF* dKO mice compared to WT mice, while *Fgf21* KO mice showed no difference in basal metabolic rate (Figure 3I,J). Substrate utilization was similar across genotypes during the assessment of basal metabolic rate (Figure 3K).

Absence of circulating GDF15/FGF21, as well as *Gdf15/Fgf21* mRNA expression in liver, was confirmed for the different KO mice examined in the HFHSD study (Figure S2A,B,D,E). Female mice had lower plasma GDF15 concentrations than male mice but similar hepatic *Gdf15* mRNA levels (Figure S2A,D). Hepatic *Gdf15* mRNA expression was elevated in male HFHSD‐fed *Fgf21* KO mice (Figure S2D). Plasma FGF21 levels were lower in female mice compared to male mice, whereas liver *Fgf21* expression was similar between sexes (Figure S2B,E).

Plasma and liver TG were similar across genotypes. Female mice had overall lower plasma TG levels than males (Figure S2C,F). Expression levels of genes associated with hepatic lipid oxidation and lipogenesis were similar across genotypes and sex (Figure S2G–O).

### 3.3 Co‐Treatment With FGF21 and GDF15 Drives Additive Weight Loss in Male Obese Mice

**FIGURE 4:**
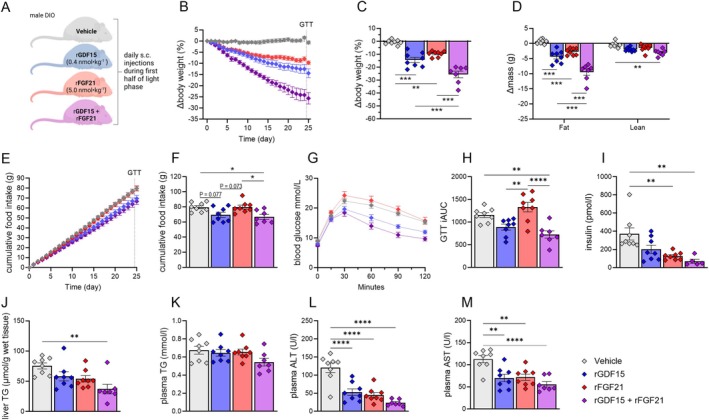
Combinatorial pharmacotherapy with rGDF15 and rFGF21 produced pronounced weight loss. (A) Male diet‐induced obese (DIO) mice received daily subcutaneous injections (5 mL/kg) of rGDF15 (0.4 nmol/kg, NNC0247‐0000‐0880), rFGF21 (5 nmol/kg, NNC0194‐0000‐0001), or rGDF15 + rFGF21 (0.4 + 5 nmol/kg), between 10.00 and 11.00 am each day for 24 days. Effect on body weight (B, C), body composition (D), and food intake were assessed. Cumulative food intake (E,F) was assessed over time. Glucose tolerance (H, I) was determined approximately 24 h after the last injections with the treatments. Plasma insulin (I), liver triacylglycerols (TG) (J), plasma TG (K), plasma alanine transaminase (ALT) (L) and plasma aspartate transaminase (AST) (M) were determined in blood and tissue collected 2 h after the last injections with treatments. Statistic: (B, F, H, I, J, L, and M) one‐way ANOVA with Tukey post hoc analyses; (D) one‐way ANOVA with fat and lean mass with Tukey post hoc analyses. *p < 0.05, **p < 0.01, ****p < 0.0001 for differences between indicated groups.

Overall, deletion of either *Gdf15,* *Fgf21,* or both had minimal effects on energy balance in male and female mice. This limited role of endogenous GDF15 and FGF21 is in juxtaposition to the pronounced effects on body weight elicited by pharmacological treatment with either factor in rodent studies. To determine whether co‐treatment with both factors provides additive benefits, we injected DIO male mice daily for 25 days with vehicle, recombinant GDF15 (0.4 nmol/kg), recombinant FGF21 (5 nmol/kg), or both (rGDF15 (0.4 nmol/kg) + rFGF21 (5 nmol/kg)) (Figure 4A). rGDF15 and rFGF21 monotherapies decreased body weight by 14.42% ± 1.99% and 9.71% ± 0.76%, respectively (Figure 4B,C). rGDF15 + rFGF21 co‐treatment induced a robust weight loss of 25.66% ± 2.55% (Figure 4B,C) that was primarily due to the loss of fat mass (Figure 4D). Food intake was lower in mice co‐treated with rGDF15 + rFGF21, with a similar trend for a decrease in food intake (*p* < 0.1) in rGDF15‐treated mice (Figure 4E,F). rFGF21 did not affect food intake (Figure 4E,F). Glucose tolerance improved in mice receiving rGDF15 alone and in the co‐treatment group (Figure 4G,H). Plasma insulin levels were lower in mice treated with rFGF21 and in mice receiving rGDF15 + rFGF21 co‐treatment (Figure 4I). Liver TG levels were approximately 50% lower following co‐treatment, while plasma TG levels remained unchanged (Figure 4J,K). Plasma levels of alanine aminotransferase (ALT) and aspartate aminotransferase (AST) were lower with all treatments relative to vehicle‐treated mice, indicating positive effects of rFGF21 and rGDF15 pharmacotherapy on liver metabolic health (Figure 4L,M).

## 4 Discussion

GDF15 and FGF21 have received significant attention over the past decade for their potential as pharmacological agents to treat obesity and type 2 diabetes. Nevertheless, their physiological role in regulating energy metabolism, including body weight, is not well understood. In this study, we demonstrate that endogenous GDF15 and FGF21 play a limited role in regulating body weight and glucose homeostasis in both male and female mice, whether fed a standard chow or a high‐fat, high‐sucrose diet. However, combined pharmacotherapy with recombinant GDF15 and FGF21 in DIO male mice resulted in significant weight loss, improved glycaemic control, and reduced liver fat, showing marked preclinical therapeutic efficacy.

The individual loss of endogenous *Gdf15* or *Fgf21* has been studied in the context of obesity. Some studies report that deleting either factor increases body weight [11, 12], while others find no effect on body weight [13, 15, 16]. Blocking GDF15 action in adult mice expressing human instead of murine *Gdf15* using a GDF15‐neutralizing antibody resulted in accelerated and significant weight gain in male mice on a high‐fat diet [23]. Whether this finding also applies to female mice remains to be studied. In a mouse model of cancer‐induced cachexia characterized by high circulating GDF15 levels and pathological weight loss, administration of a GDF15‐neutralizing antibody induced pronounced weight regain in female mice; male mice were not assessed [24]. Collectively, these data suggest that compensatory mechanisms associated with the lifelong absence of a gene might mask its physiological function, since the effects of blocking GDF15 in adult mice appear more robust.

Compensation between FGF21 and GDF15 may occur, with one compensating for the other due to their similar molecular regulation and overlapping functions. However, our findings show only minimal effects due to the combined deletion of *Gdf15* or *Fgf21*, and these effects were limited to male mice on a HFHSD. Notably, these trends appeared to be driven by the absence of GDF15, contradicting the hypothesis of additive deleterious effects from the combined knockout. The male‐specific effects align with previous findings by Tran et al. [11], who reported no difference in body weight between female *Gdf15* knockout and wild‐type mice, regardless of diet. This underscores the need to consider sex‐specific differences in GDF15 biology.

The trend of increased body weight in male *Gdf15* KO and *GF* dKO mice on HFHSD was not due to changes in daily energy expenditure. This is consistent with the unchanged energy expenditure observed in DIO *Gdf15*‐null mice reported by Chung et al. [15], but in contrast to another study reporting lower energy expenditure in high‐fat diet‐fed *Gdf15* KO mice with increased body weight [11]. Interestingly, the basal metabolic rate was elevated in both DIO *Gdf15* KO and DIO *GF* dKO mice, even when corrected for the 4–5 g difference in body weight by ANCOVA analyses. The reason for this increase requires further investigation.

Patel and colleagues recently investigated the combined loss of GDF15 and FGF21 in a double knockout model, focusing on male mice fed a high‐fat, moderate‐sucrose diet [25]. They observed a moderate increase in weight gain due to the loss of GDF15, consistent with the male‐specific effect we obeserved a trend for. Notably, we show that this weight gain effect is absent in female mice. Together, our findings and those of Patel and colleagues appear to reject the hypothesis that endogenous FGF21 and GDF15 together provide significant resistance to DIO. Moreover, the combined loss of FGF21 and GDF15 had no impact on weight gain in chow‐fed mice.

In our study, the loss of endogenous GDF15 or FGF21 had no effect on glucose tolerance in mice fed either a chow, or high‐fat, high‐sucrose diet. This contrasts with previous studies that reported glucose intolerance in *Fgf21* KO mice on a chow diet, but not on a high‐fat diet [16]. Additionally, while *Gdf15* KO mice have been reported as glucose intolerant [11], other studies found no differences in glucose tolerance [15]. Our findings show that the absence of both GDF15 and FGF21 had no negative impact on glucose tolerance in either male or female mice, suggesting that endogenous levels of these factors are not essential for regulating glucose homeostasis.

In contrast to the minimal effects of endogenous FGF21 and GDF15 on body weight and glycemia, our study demonstrates that combined pharmacotherapy with rGDF15 and rFGF21 induces significant weight loss. This weight loss appears to result from reduced food intake, primarily driven by rGDF15, and potentially increased energy expenditure, though this was not directly studied. Furthermore, rFGF21 may contribute to an increase in metabolic rate through adipose tissue browning [12, 17, 26], while rGDF15 might enhance energy expenditure via muscle calcium futile cycling [18]. Future studies should determine whether the weight loss reflects heightened energy expenditure and, using hypothalamic c‐Fos mapping after combined rFGF21 + rGDF15 treatment, establish whether the hormones engage shared or distinct feeding circuits.

FGF21 and its analogues are currently being investigated in clinical trials for the treatment of obesity, type 2 diabetes, and fatty liver disease [27, 28], while the clinical potential of rGDF15 for obesity treatment is also being explored [29]. However, the efficacy of rFGF21 in promoting weight loss in individuals with obesity has been underwhelming [28], and it has instead gained attention as a treatment for fatty liver disease [27]. Limited clinical data exist for rGDF15, but a phase 1 study showed that a GDF15 analogue modestly reduced weight by decreasing appetite, but dose‐dependent nausea and vomiting were also observed [29]. Our data suggest an opportunity: combining rFGF21 and rGDF15 could target appetite (rGDF15) thereby promoting weight loss, while also correcting fatty liver (rFGF21). Potential synergistic effects between rGDF15 and rFGF21 could also allow for reduction in the rGDF15 dose thereby avoiding nausea‐related side effects, while in conjunction with rFGF21 still eliciting metabolic benefits.

While previous research has shown that pharmacotherapy with rGDF15, and to a lesser extent rFGF21, can improve glycemia, our study found improved glucose tolerance only with rGDF15 monotherapy and combination therapy. This result may be influenced by the fact that rGDF15 has a longer half‐life than rFGF21 combined with the timing of the glucose tolerance (≈24 h after last injection). Indeed, we have previously reported high exposure of rGDF15 but undetectable rFGF21 exposure in mice ≈24 h after last injection. However, rFGF21 did reduce circulating insulin levels, suggesting improved insulin sensitivity even though overall glucose regulation remained unchanged.

Our study has limitations. Our phenotyping of the loss‐of‐function models is underpowered, but it can be concluded that our sample size is sufficient to demonstrate that the combined deletion does not lead to any dramatic changes in body weight gain that would be easily detectable. It is also possible that the effects on energy balance and glucose homeostasis of deleting GDF15, FGF21, or both would become more apparent in older mice, with levels known to rise even more with aging. The mouse background strain (see Section 2) may also contribute to our observed results because strain differences are known to affect energy‐balance outcomes in response to diet interventions [30]. Our pharmacology results will also have to be assessed in female obese mice, but generally the male obese C57BL/6 mouse better captures common obesity in humans with concomitant insulin resistance, whereas female C57BL/6 mice are more resistant to DIO and often remain insulin sensitive throughout. Our DIO results were obtained using diets that combined high fat with substantial amounts of sucrose, a regimen chosen because it maximizes weight gain in C57BL/6 mice [31]. Whether the same outcomes would arise with high fat or high sucrose alone remains to be tested. It is still unclear whether the metabolic benefits observed with combined rFGF21 and rGDF15 treatment are intrinsic to the hormones per se or secondary to the pronounced weight loss they induce.

In conclusion, we show that endogenous FGF21 and GDF15 do not confer resistance to DIO in male and female mice, while pharmacological co‐treatment with rFGF21 and rGDF15 produces sizable weight loss.

## Author Contributions

M.K., K.J., R.E.K., C.C. conceived and designed the study. K.J., S.E., J.M.J, L.D., E.P., A.B.‐R. contributed to data collection. K.J. and S.E. contributed to data analyses and interpretation. K.J., S.E., and M.K. contributed to writing the manuscript. M.K. is the guarantor of this work and, as such, had full access to all the data in the study and takes responsibility for the integrity of the data and the accuracy of the data analysis.

## Conflicts of Interest

C.C. is co‐founder of Ousia Pharma ApS, a biotech company developing therapeutics for obesity. J.M.J. and R.E.K. are employed by Novo Nordisk A/S, and some are minor shareholders in Novo Nordisk A/S. The remaining authors declare no competing interests.

## Supporting information


**Figure S1:** Plasma GDF15 (A) plasma FGF21 (B), plasma and liver triacylglycerol (TG) (C, F), and mRNA abundance (D, E, G–O) of indicated genes in livers from chow‐fed male and female wild‐type (WT), Gdf15 knockout (KO), Fgf21 KO, and Gdf15 × Fgf21 (GF) double KO (dKO) mice. All data were analysed with two‐way (genotype × sex) ANOVA with Tukey post‐hoc testing when an interaction was detected.
**Figure S2:** Gene expression in HSHFD. Plasma GDF15 (A) plasma FGF21 (B), plasma and liver triacylglycerol (TG) (C, F), and mRNA abundance (D, E, G–O) of indicated genes in livers from HFHSD‐fed male and female wild‐type (WT), Gdf15 knockout (KO), Fgf21 KO, and Gdf15 × Fgf21 (GF) double KO (dKO) mice. All data were analysed with two‐way (genotype × sex) ANOVA with Tukey post‐hoc testing when an interaction was detected.

## Data Availability

The data that support the findings of this study are available in Sections 2 and 3, and/or Figures S1 and S2 of this article.
