# Supplementary material for: Pharmacological but Not Physiological Levels of GDF15 and FGF21 Regulate Body Weight and Glycemic Control in Obese Mice
Source: FASEB J. 2025 Aug 11;39(15):e70918. doi: 10.1096/fj.202501350R (PMC12337615; doi:10.1096/fj.202501350R)
Supplement: Supplementary file 1 — Figure S1: Plasma GDF15 (A) plasma FGF21 (B), plasma and liver triacylglycerol (TG) (C, F), and mRNA abundance (D, E, G–O) of indicated genes in livers from chow‐fed male and female wild‐type (WT), Gdf15 knockout (KO), Fgf21 KO, and Gdf15 × Fgf21 (GF) double KO (dKO) mice. All data were analysed with two‐way (genotype × sex) ANOVA with Tukey post‐hoc testing when an interaction was detected. Figure S2: Gene expression in HSHFD. Plasma GDF15 (A) plasma FGF21 (B), plasma and liver triacylglycerol (TG) (C, F), and mRNA abundance (D, E, G–O) of indicated genes in livers from HFHSD‐fed male and female wild‐type (WT), Gdf15 knockout (KO), Fgf21 KO, and Gdf15 × Fgf21 (GF) double KO (dKO) mice. All data were analysed with two‐way (genotype × sex) ANOVA with Tukey post‐hoc testing when an interaction was detected. [file FSB2-39-e70918-s001.pdf]

Supplementary Online Info

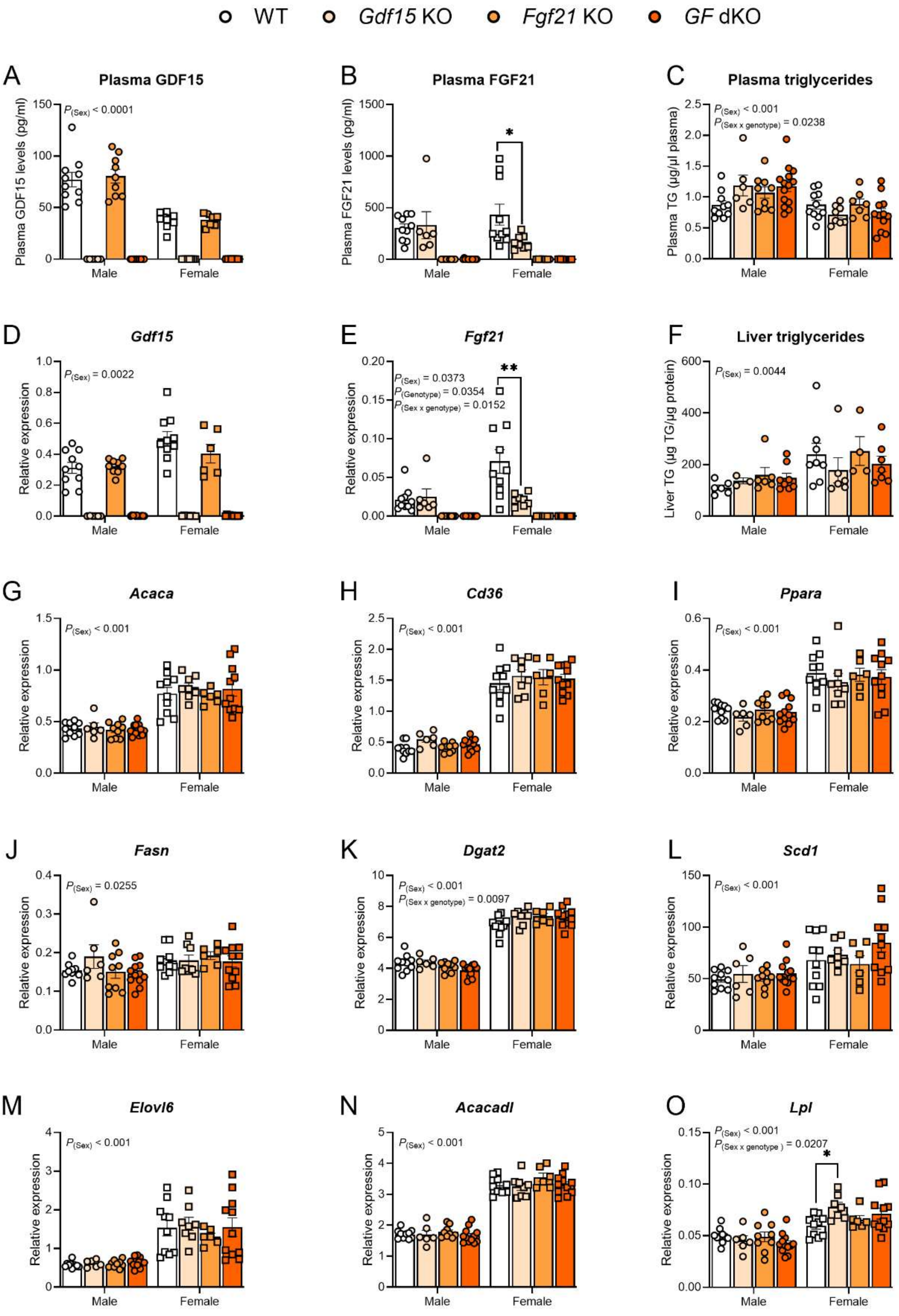

Supplementary Figure 1. Plasma GDF15 (A) plasma FGF21 (B), plasma and liver triacylglycerol (TG) (C,F), and mRNA abundance (D,E,G-O) of indicated genes in livers from chow-fed male and female wildtype (WT), *Gdf15* knockout (KO), *Fgf21* KO, and Gdf15 x Fgf21 (*GF*) double KO (dKO) mice. All data were analysed with two-way (genotype x sex) ANOVA with Tukey post-hoc testing when an interaction was detected.

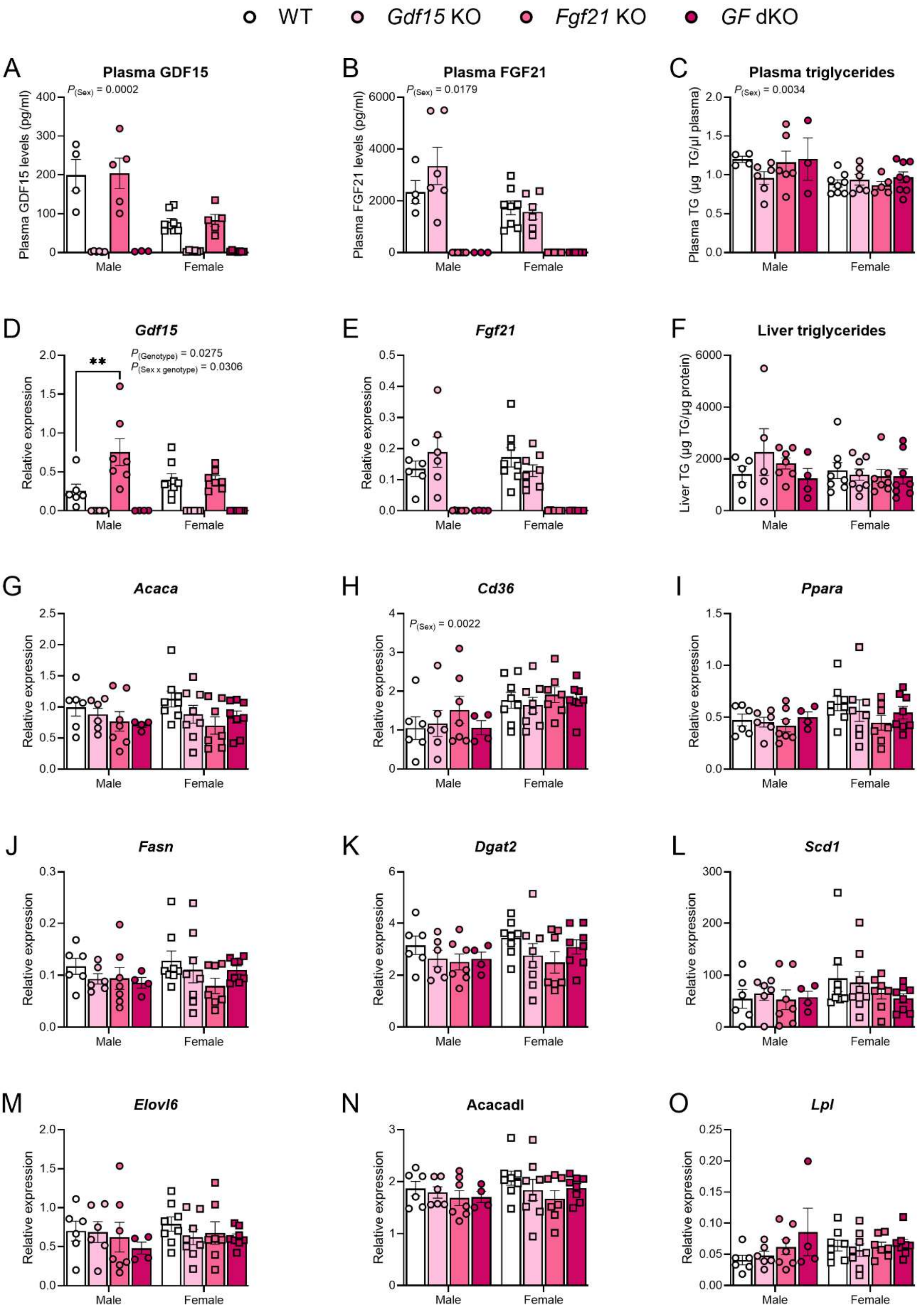

Supplementary Figure 2. Gene expression in HSHFD. Plasma GDF15 (A) plasma FGF21 (B), plasma and liver triacylglycerol (TG) (C,F), and mRNA abundance (D,E,G-O) of indicated genes in livers from HFHSD-fed male and female wildtype (WT), *Gdf15* knockout (KO), *Fgf21* KO, and Gdf15 x Fgf21 (*GF*) double KO (dKO) mice. All data were analysed with two-way (genotype x sex) ANOVA with Tukey post-hoc testing when an interaction was detected.
